# Geographical Living Settings, Lifestyle Behaviors, and the Prevalence of Prediabetes or Diabetes: A Cross-Sectional Community-Based Study in Northern Ibaraki, Japan

**DOI:** 10.7759/cureus.111868

**Published:** 2026-07-01

**Authors:** Constanza I Bravo Cabrera, Yukiko Wagatsuma, Urme Binte Sayeed

**Affiliations:** 1 Department of Clinical Trial and Clinical Epidemiology, Graduate School of Comprehensive Human Sciences, University of Tsukuba, Tsukuba, JPN; 2 Department of Clinical Trial and Clinical Epidemiology, Faculty of Medicine, University of Tsukuba, Tsukuba, JPN

**Keywords:** diabetes, geographical living settings, health-related behaviors, prediabetes, suburban environments

## Abstract

Diabetes mellitus is a growing public health concern worldwide, shaped by both individual and environmental factors. This cross-sectional study looked at how geographical living settings relate to the prevalence of prediabetes or diabetes among 1880 participants in northern Ibaraki, Japan, of whom 1255 (66.8%) had prediabetes or diabetes. Participants were grouped as urban, suburban, or rural based on population density. Glycemic status was measured directly through HbA1c during community health examinations, and lifestyle information was gathered through standardized self-reported questionnaires. Prediabetes and diabetes were combined into a single category for the analysis. After adjusting for age, sex, BMI, and selected lifestyle behaviors, suburban participants had lower odds of prediabetes or diabetes than urban participants (OR = 0.69, 95% CI 0.54-0.89, p = 0.005), while the rural setting showed no significant association. Both daily and occasional alcohol consumption were linked to lower odds compared with rarely drinking, and lifestyle behaviors differed by sex and geographical living setting. Overall, these findings suggest that where people live may be connected to their odds of having prediabetes or diabetes, and they point to the value of considering suburban areas as their own category rather than relying on a simple urban-rural division. As a cross-sectional study, the results show associations rather than cause and effect, and would need to be confirmed in prospective studies. These findings may also help guide future public health efforts in Japan and similar regional settings, where suburban settings may offer a mix of features that support better glycemic status and deserve closer attention.

## Introduction

Diabetes mellitus is a chronic metabolic disorder characterized by elevated blood glucose levels and represents a major global public health concern across diverse populations around the world. In 2024, an estimated 11.1% of adults aged 20-79 years (589 million people) were living with diabetes worldwide, a proportion projected to reach approximately 13% (853 million) by 2050 [1]. Prevalence is consistently higher in urban (12.3%) than in rural (9.2%) areas, accentuating the relevance of the living environment to metabolic risk [1]. In Japan, the National Health and Nutrition Survey of 2019 found that 19.7% of men and 10.8% of women aged ≥20 years were strongly suspected of having diabetes [2], while age-standardized estimates from earlier national surveys (2003 to 2012) placed adult diabetes prevalence at approximately 8%, with higher rates in men and in adults aged ≥65 years [3]. Prevalence of prediabetes and diabetes represents a growing public health challenge engaging healthcare systems, economies, and the well-being of populations [4]. In Japan, the increasing impacts of diabetes have been driven by the aging population and changing lifestyles, positioning early identification of risk factors and location of population at risk as a priority [5].

While individual-level factors, such as age, sex, genetic predisposition, body mass index (BMI), and health-related behaviors, are well-established contributors to the risk of developing diabetes, growing attention is being given to the role of environmental and circumstantial factors, including the physical and social characteristics of the living environment [6,7]. Specifically, geographical and urban-rural living settings may influence the risk of metabolic disorders by determining access to healthcare services, availability of nutritious food, opportunities for physical activity, social networks, and exposure to environmental stressors or pollutants [8,9].

The association and effect of geographical living settings on the occurrence and risk of prediabetes and diabetes, nevertheless, remains mixed across studies and populations. Research from South Asian countries has shown that rural populations tend to have lower prevalence of diabetes than densely populated areas [10,11], likely due to differences in dietary habits, physical activity, and occupational patterns; however, regional prevalence is still high overall and rising with urbanization [12]. In contrast, studies from the United States have indicated higher rates of diabetes and related risk factors in rural populations, potentially attributable to socioeconomic disparities, limited healthcare access, and reduced health literacy [9,13]. These varying conclusions emphasize the need to investigate how regional and cultural contexts interact with environmental and lifestyle factors to influence prediabetes and diabetes occurrence.

In Japan, most research on the geographical distribution of diabetes has focused on broad rural-urban differences [14,15], with limited understanding of suburban areas, which may offer a distinctive mix of features from both rural and urban communities. Suburban settings could serve as a model for living environments conducive to health, yet their relationship with chronic disease prevalence remains underexplored. Despite Japan's high level of urbanization [16], suburban and rural areas remain comparatively understudied, even though neighborhood environments are known to influence the occurrence of diabetes [17].

Health-related lifestyle behaviors, such as dietary behaviors and physical activity, have been linked to diabetes risk and are often conditioned by cultural patterns and opportunities provided by local environments [18,19]. The interaction between health-related lifestyle behaviors and living settings may further explain geographical disparities in the prevalence of prediabetes and diabetes, justifying their inclusion in studies aiming to understand the determinants of diabetes in specific populations.

Given the complexity and variability of these influences, a better understanding of how geographical living settings interact with sociodemographic factors and lifestyle behaviors to shape the prevalence of prediabetes or diabetes is needed to design effective public health interventions. The present study aimed to investigate the association between geographical living settings and the prevalence of prediabetes or diabetes and to examine how lifestyle behaviors, including exercise and alcohol consumption, relate to these conditions by using data from a community-based cohort in Ibaraki, Japan, in which residents attend annual health examinations organized in partnership with Japan Agriculture Cooperative of Ibaraki (JA Ibaraki) [20]. Given the cross-sectional design, the findings should be interpreted as observational associations rather than causal or directional effects. By including suburban settings as a distinct category, this research contributes to a broader understanding of the distribution of prediabetes or diabetes within a localized Japanese setting.

## Materials and methods

Study area and study participants

This study was conducted in Mito and the surrounding areas of Ibaraki prefecture, Japan. Mito is located approximately 125 km north of Tokyo. The study was embedded in an ongoing community-based cohort study designed to contribute to health knowledge and promote health improvement in the community. Participants were invited through annual health examinations conducted at a regional hospital in Mito or at its outreach sites, organized in partnership with JA Ibaraki [20]. In general, approximately 5000 individuals attend these examinations annually, about half of them at outreach sites. Of the 4014 participants examined across 2019, the 1880 who attended an outreach site were included in this analysis. Examinations were carried out by healthcare staff at 23 outreach sites, and the information was collected by trained research staff from the data center at the regional hospital.

Study design

This was a cross-sectional study based on data from health examinations conducted at 23 outreach sites in northern Ibaraki during 2019. Examinations were performed on different dates across the year. A single examination per participant was retained, so each participant contributed to one observation. 

Participant allocation and geographical classification

Participants were allocated into nine areas based on the date and place of the outreach examination attended during 2019, used as an indicator of area of residence. The outreach examinations were performed at the community level and held at local sites, so it was assumed that residents generally attended the examination site located in or nearest to their own community. This supports the use of the outreach examination location as an indicator of area of residence, considering that residential address was not available in the anonymized dataset used for this study.

Classification of the nine areas into three geographical living settings was done by population density as inhabitants/km²: rural as less than 125/km², suburban as 125 to 999/km², and urban as 1000/km² or more [21,22]. Considering no single urban or rural definition is suitable for all settings [22], the thresholds were adapted to the relatively low population density of the study region so that the rural, suburban, and urban categories were each represented. Population density data were obtained from the 2015 Population Census of Japan (Statistics Bureau, Ministry of Internal Affairs and Communications) [23], the most recent national census preceding the 2019 examinations. As the population of the study region changes only gradually, these figures were considered representative of the 2019 study period.

Measurements and data collection

Data were collected during the 2019 annual health examinations at the 23 outreach sites. Demographic information and examination measurements were obtained by trained healthcare staff at each site. BMI was calculated as weight divided by height squared (kg/m²). Blood pressure and anthropometric measurements, including waist circumference, were taken on site. Glycated hemoglobin (HbA1c) was analyzed at the regional hospital laboratory. Prediabetes was defined as HbA1c of 5.6 to 6.4% and diabetes as HbA1c of 6.5% or higher or current use of anti-diabetic medication. For the analysis, prediabetes and diabetes were combined into a single category, defined as an HbA1c of 5.6% or higher or current use of anti-diabetic medication. Participants were not subclassified, so this category represents the combined presence of prediabetes or diabetes. Lifestyle information was obtained using a standardized self-administered questionnaire for chronic disease control specified by the Ministry of Health, Labour and Welfare of Japan (MHLW) onsite. From this questionnaire, lifestyle variables previously used in studies based on this cohort [20,24] were selected, covering smoking, alcohol consumption, exercise, dietary habits, and sleep. The questionnaire and response criteria are provided in Appendix 1.

Statistical analysis

Descriptive statistics were used to review general characteristics of the participants. Means and standard deviations (SDs) were described for continuous variables, whereas frequencies and percentages were presented for categorical variables. Comparisons among sex and geographical living settings were performed using one-way ANOVA for continuous variables and chi-square tests for categorical variables. Descriptive comparisons of health-related lifestyle behaviors by sex and by geographical living setting were also performed to further characterize the study population, using chi-square tests.

Binary logistic regression analyses were conducted to examine the association between geographical living settings and the prevalence of prediabetes or diabetes. Multivariable models were constructed considering the dependent variable as the combined prediabetes or diabetes category. The main explanatory variable was the geographical living setting (rural, suburban, urban). Age, sex, BMI, and health-related lifestyle behaviors were included as covariates across the adjusted models. Model 3, adjusted for age, sex, BMI, and health-related lifestyle behaviors, was the fully adjusted model. Models 1 and 2 are presented to show the effect of progressive adjustment: Model 1 was adjusted by age and sex, while Model 2 was adjusted by age, sex, and BMI. Sensitivity analyses were performed to assess the robustness of the findings. As prediabetes and diabetes were analyzed as a single combined category, each condition was additionally modeled separately against the normoglycemic group. The robustness of the associations was further assessed by additionally adjusting for hypertension. These analyses are presented in the Appendix 1 to Appendix 4. All tests were two-sided. Odds ratios (ORs) with 95% confidence intervals (CIs) were calculated. A p-value of less than 0.05 was considered significant. No adjustment for multiple comparisons was applied. Missing data were handled by complete-case analysis, and no imputation was performed. All statistical analyses were conducted using IBM SPSS Statistics for Windows, Version 30.0 (IBM Corp., Armonk, NY).

## Results

General characteristics of the 1880 participants by three geographical living settings are presented in Table 1. The largest proportion of participants were from urban areas (880, 46.8%), followed by suburban (601, 32.0%), and rural areas (399, 21.2%).

**Table 1:** General characteristics of study participants. Abbreviations: BMI, body mass index; df, degrees of freedom; F, one-way ANOVA F statistic; χ², chi-square test. ^a^Obtained from the 2015 Population Census of Japan (Statistics Bureau, Ministry of Internal Affairs and Communications); values shown are the mean of the area densities within each group. ^b^Glycemic status compared by omnibus chi-square test (header row); category comparisons shown for each subgroup. ^c^Prediabetes only: HbA1c ≥5.6% to 6.4% and not taking anti-diabetic medication. ^d^Diabetes only: HbA1c ≥6.5% or taking anti-diabetic medication. ^e^Combined category of prediabetes or diabetes: HbA1c ≥5.6% or taking anti-diabetic medication; used in the regression models. ^f^Chi-square test was used for categorical variables, whereas the one-way ANOVA F statistic was used for continuous variables. *p < 0.05.

Characteristics	Overall, n = 1880	Urban area, n = 880	Suburban area, n = 601	Rural area, n = 399	Test statistics^f^	df	p-value
								Age, years ± SD	50.0 ± 16.9	49.7 ± 17.2	51.4 ± 16.8	48.8 ± 16.4	F = 3.37	2	0.035*
								Age, years (min-max)	18-92	18-89	18-92	18-90	-	-	-
Age group					χ² = 17.15	4	0.002*
<40 years, n (%)	594 (31.6)	304 (34.5)	161 (26.8)	129 (32.3)	-	-	-
40-64 years, n (%)	846 (45.0)	360 (40.9)	292 (48.6)	194 (48.6)	-	-	-
≥65 years, n (%)	440 (23.4)	216 (24.5)	148 (24.6)	76 (19.0)	-	-	-
Sex					χ² = 9.99	2	0.007*
Males, n (%)	947 (50.4)	426 (48.4)	292 (48.6)	229 (57.4)	-	-	-
Females, n (%)	933 (49.6)	454 (51.6)	309 (51.4)	170 (42.6)	-	-	-
BMI, mean ± SD	23.5 ± 4.0	23.4 ± 3.9	23.5 ± 4.0	23.8 ± 4.1	F = 1.33	2	0.264
Population density^a^, population/km^2^	460.6	1402	338.2	81.8	-	-	-
Hypertension, n (%)	881 (46.9)	391 (44.4)	301 (50.1)	189 (47.4)	χ² = 4.54	2	0.103
Glycemic status^b^, n (%)					χ² = 4.91	4	0.297
Normoglycemia	625 (33.2)	275 (31.3)	209 (34.8)	141 (35.3)	-	-	-
Prediabetes only^c,^ n (%)	1072 (57.0)	518 (58.9)	328 (54.6)	226 (56.6)	χ² = 2.71	2	0.258
Diabetes only^d^, n (%)	183 (9.7)	87 (9.9)	64 (10.6)	32 (8.0)	χ² = 1.93	2	0.381
Prediabetes or diabetes combined^e^, n (%)	1255 (66.8)	605 (68.8)	392 (65.2)	258 (64.7)	χ² = 3.13	2	0.21

The mean age of the participants was 50.0 years (SD = 16.9) and differed across geographical living settings (p = 0.035), with suburban participants presenting the highest overall age (mean = 51.4, SD = 16.8) and rural participants the youngest (mean = 48.8, SD = 16.4). The distribution of age groups also differed across settings (p = 0.002). Overall, 594 (31.6%) participants were younger than 40 years, 846 (45.0%) were 40-64 years, and 440 (23.4%) were 65 years or older.

Males comprised 947 (50.4%) of participants, and the proportion of males also differed across living settings (p = 0.007); a larger proportion was present in rural areas (229, 57.4%). BMI was comparable across living settings (overall mean = 23.5, SD = 4.0; p = 0.264). Population density was highest in urban settings (1402.0/km²) and lowest in rural settings (81.8/km²).

Participants with hypertension constituted 46.9% overall, with no significant difference across living settings (p = 0.103). Regarding glycemic status, 1072 (57.0%) participants had prediabetes, and 183 (9.7%) had diabetes. The combined prevalence of prediabetes or diabetes was 66.8% overall and did not differ significantly across living settings (68.8% in urban, 65.2% in suburban, and 64.7% in rural areas; p = 0.21).

To further characterize the study population, differences in health-related lifestyle behaviors were examined by sex (Table 2) and by geographical living setting (Table 3).

**Table 2:** Health-related lifestyles by sex. Abbreviation: df, degrees of freedom. ^a^Missing, n = 1; ^b^missing, n = 2; ^c^missing, n = 9; ^d^missing, n = 1; ^e^missing, n = 9; ^f^missing, n = 2; ^g^missing, n = 10; ^h^missing, n = 5; ^i^missing, n = 4; ^j^missing, n = 23. * p < 0.05.

Lifestyles	Overall	Male	Female	Chi-square test (χ²)	df	p-value
							Smoking^a^, n (%)				235.33	1	<0.001*
Yes	446 (23.7)	366 (38.7)	80 (8.6)	-	-	-
No	1433 (76.3)	580 (61.3)	853 (91.4)	-	-	-
Regular exercise^b^, n (%)				10.23	1	0.001*
Yes	412 (21.9)	236 (25.0)	176 (18.9)	-	-	-
No	1466 (78.1)	709 (75.0)	757 (81.1)	-	-	-
Alcohol consumption^c^, n (%)				205.21	2	<0.001*
Daily	358 (19.1)	291 (30.9)	67 (7.2)	-	-	-
Occasionally	483 (25.8)	262 (27.8)	221 (23.8)	-	-	-
Rarely	1030 (55.1)	389 (41.3)	641 (69.0)	-	-	-
Daily walking^d^, n (%)				1.82	1	0.178
Yes	634 (33.7)	333 (35.2)	301 (32.3)	-	-	-
No	1245 (66.3)	613 (64.8)	632 (67.7)	-	-	-
Walk faster than peers^e^, n (%)				0.05	1	0.827
Yes	707 (37.8)	359 (38.0)	348 (37.5)	-	-	-
No	1164 (62.2)	585 (62.0)	579 (62.5)	-	-	-
Eating speed^f^, n (%)				46.38	2	<0.001*
Fast	496 (26.4)	312 (33.0)	184 (19.7)	-	-	-
Normal	1203 (64.1)	563 (59.6)	640 (68.6)	-	-	-
Slow	179 (9.5)	70 (7.4)	109 (11.7)	-	-	-
Late dinner^g^, n (%)				73.79	1	<0.001*
Yes	542 (29.0)	357 (37.9)	185 (19.9)	-	-	-
No	1328 (71.0)	584 (62.1)	744 (80.1)	-	-	-
Eating snacks^h,^ n (%)				57.55	2	<0.001*
Everyday	301 (16.1)	112 (11.9)	189 (20.3)	-	-	-
Sometimes	1181 (63.0)	576 (61.0)	605 (65.1)	-	-	-
Rarely	393 (21.0)	257 (27.2)	136 (14.6)	-	-	-
Skipping breakfast^i^, n (%)				54.21	1	<0.001*
Yes	371 (19.8)	250 (26.5)	121 (13.0)	-	-	-
No	1505 (80.2)	693 (73.5)	812 (87.0)	-	-	-
Adequate sleep^j^, n (%)				2.88	1	0.090
Yes	1319 (71.0)	680 (72.8)	639 (69.2)	-	-	-
No	538 (29.0)	254 (27.2)	284 (30.8)	-	-	-

**Table 3:** Health-related lifestyles by area. Abbreviations: df, degrees of freedom. ^a^Missing, n = 1; ^b^missing, n = 2; ^c^missing, n = 9; ^d^missing, n = 1; ^e^missing, n = 9; ^f^missing, n = 2; ^g^missing, n = 10; ^h^missing, n = 5; ^i^missing, n = 4; ^j^missing, n = 23. *p < 0.05.

Lifestyles	Overall	Urban area	Suburban area	Rural	Chi-square test (χ²)	df	p-value
								Smoking^a^, n (%)					20.7	2	<0.001*
Yes	446 (23.7)	187 (21.3)	130 (21.6)	129 (32.3)	-	-	-
No	1433 (76.3)	692 (78.7)	471 (78.4)	270 (67.7)	-	-	-
Regular exercise^b^, n (%)					8.14	2	0.017*
Yes	412 (21.9)	214 (24.4)	109 (18.1)	89 (22.3)	-	-	-
No	1466 (78.1)	664 (75.6)	492 (81.9)	310 (77.7)	-	-	-
Alcohol consumption^c^, n (%)					9.87	4	0.043*
Daily	358 (19.1)	150 (17.1)	114 (19.2)	94 (23.6)	-	-	-
Occasionally	483 (25.8)	246 (28.1)	143 (24.0)	94 (23.6)	-	-	-
Rarely	1030 (55.1)	481 (54.8)	338 (56.8)	211 (52.9)	-	-	-
Daily walking^d^, n (%)					3.21	2	0.201
Yes	634 (33.7)	302 (34.4)	187 (31.1)	145 (36.3)	-	-	-
No	1245 (66.3)	577 (65.6)	414 (68.9)	254 (63.7)	-	-	-
Walks faster than peers^e^, n (%)					1.81	2	0.405
Yes	707 (37.8)	346 (39.4)	219 (36.7)	142 (35.9)	-	-	-
No	1164 (62.2)	533 (60.6)	378 (63.3)	253 (64.1)	-	-	-
Eating speed^f^, n (%)					7.06	4	0.133
Fast	496 (26.4)	218 (24.8)	154 (25.7)	124 (31.1)	-	-	-
Normal	1203 (64.1)	571 (65.0)	387 (64.5)	245 (61.4)	-	-	-
Slow	179 (9.5)	90 (10.2)	59 (9.8)	30 (7.5)	-	-	-
Late dinner^g^, n (%)					1.89	2	0.388
Yes	542 (29.0)	265 (30.4)	162 (27.0)	115 (28.9)	-	-	-
No	1328 (71.0)	608 (69.6)	437 (73.0)	283 (71.1)	-	-	-
Eating snacks^h^, n (%)					0.5	4	0.973
Everyday	301 (16.1)	137 (15.6)	100 (16.7)	64 (16.1)	-	-	-
Sometimes	1181 (63.0)	559 (63.7)	372 (62.0)	250 (62.8)	-	-	-
Rarely	393 (21.0)	181 (20.6)	128 (21.3)	84 (21.2)	-	-	-
Skipping breakfast^i^, n (%)					11.24	2	0.004*
Yes	371 (19.8)	169 (19.2)	101 (16.9)	101 (25.4)	-	-	-
No	1505 (80.2)	710 (80.8)	498 (83.1)	297 (74.6)	-	-	-
Adequate sleep^j^, n (%)					10.18	2	0.006*
Yes	1319 (71.0)	620 (71.1)	395 (67.2)	304 (76.6)	-	-	-
No	538 (29.0)	252 (28.9)	193 (32.8)	93 (23.4)	-	-	-

Table 2 shows health-related lifestyles by sex. Males showed a higher proportion of smoking than females (366, 38.7% vs. 80, 8.6%; p < 0.001) and were more likely to report regular exercise (236, 25.0% vs. 176, 18.9%; p = 0.001). Alcohol consumption also differed between sexes as 291 (30.9%) of males reported daily consumption compared with 67 (7.2%) of females, in comparison with the greater proportion of females who reported rarely consuming alcohol (641, 69.0% vs. 389, 41.3%; p < 0.001).

Eating behaviors differed by sex as well. Males more often reported fast eating speed (312, 33.0% vs. 184, 19.7%), whereas females more often reported slow eating (109, 11.7% vs. 70, 7.4%; p < 0.001). Late dinner habit was more frequent among males (357, 37.9% vs. 185, 19.9%; p < 0.001), while daily snacking was more common among females (189, 20.3% vs. 112, 11.9%; p < 0.001). No significant differences between males and females were observed for daily walking, walking faster than peers, or adequate sleep.

Health-related lifestyle behaviors across geographical living settings are shown in Table 3. Smoking was more prevalent in rural areas (129, 32.3%) than in urban (187, 21.3%) and suburban areas (130, 21.6%; p < 0.001). Daily alcohol consumption was also highest in rural settings (94, 23.6%) and lowest in urban areas (150, 17.1%; p = 0.043), while occasional consumption was the highest in urban areas (246, 28.1%). Regular exercise was most prevalent in urban areas (214, 24.4%) and least in suburban areas (109, 18.1%; p = 0.017). No significant differences across settings were found for daily walking, walking faster than peers, eating speed, late dinner, or snacking. Among the lifestyle behaviors examined, alcohol consumption and regular exercise were subsequently included in the multivariable models examining the association between living setting and prediabetes or diabetes.

The associations between geographical living setting and the prevalence of prediabetes or diabetes are presented in Tables 4-6.

**Table 4:** Model 1. Association between geographical living setting and prevalence of prediabetes or diabetes (n = 1880), adjusted by age and sex. Abbreviations: CI, confidence intervals; df, degrees of freedom; F, Wald F test; OR, odds ratio; χ², Wald chi-square test. ^a^Wald chi-square was used for categorical variables, whereas Wald F was used for continuous variables. *p < 0.05.

Variables	OR	95% CI	Test statistic^a^	df	p-value
Area					
Urban	Reference				
Suburban	0.729	0.572-0.931	χ² = 6.45	1	0.011*
Rural	0.813	0.618-1.069	χ² = 2.19	1	0.139
Age (continuous)	1.063	1.055-1.070	F = 268.55	1	0.000*
Sex					
Female	Reference				
Male	1.271	1.022-1.581	χ² = 4.64	1	0.031*

**Table 5:** Model 2. Association between geographical living setting and prevalence of prediabetes or diabetes (n = 1880), adjusted by age, sex and BMI. Abbreviations: CI, confidence intervals; df, degrees of freedom; F, Wald F test; OR, odds ratio; χ², Wald chi-square test. ^a^Wald chi-square was used for categorical variables, whereas Wald F was used for continuous variables. *p < 0.05.

Variables	OR	95% CI	Test statistic ^a^	df	p-value
Area					
Urban	Reference				
Suburban	0.698	0.543-0.897	χ² = 7.90	1	0.005*
Rural	0.761	0.574-1.008	χ² = 3.62	1	0.057
Age (continuous)	1.062	1.054-1.070	F = 252.61	1	0.000*
Sex					
Female	Reference				
Male	0.976	0.775-1.229	χ² = 0.043	1	0.836
BMI (continuous)	1.140	1.106-1.175	F = 71.58	1	0.000*

**Table 6:** Model 3. Association between geographical living setting and prevalence of prediabetes or diabetes (n = 1880), adjusted by age, sex, BMI, alcohol consumption (n = 1870), and regular exercise (n = 1870). Abbreviations: CI, confidence intervals; df, degrees of freedom; F, Wald F test; OR, odds ratio; χ², Wald chi-square test. ^a^Wald chi-square was used for categorical variables, whereas Wald F was used for continuous variables. ^b^Model 3: Alcohol consumption, n = 1870; regular exercise, n = 1870. *p < 0.05.

Variables	OR	95% CI	Test statistic^a^	df	p-value
Area					
Urban	Reference				
Suburban	0.694	0.538-0.895	χ² = 7.89	1	0.005*
Rural	0.767	0.577-1.019	χ² = 3.35	1	0.067
Age (continuous)	1.064	1.056-1.072	F = 240.78	1	0.000*
Sex					
Female	Reference				
Male	1.165	0.907-1.496	χ² = 1.43	1	0.232
BMI (continuous)	1.140	1.105-1.176	F = 68.02	1	0.000*
Alcohol consumption^b^					
Rarely	Reference				
Occasionally	0.661	0.509-0.858	χ² = 9.65	1	0.002*
Daily	0.473	0.347-0.643	χ² = 22.78	1	0.000*
Regular exercise^b^					
No	Reference				
Yes	1.190	0.896-1.580	χ² = 1.44	1	0.230

In Model 1 (Table 4), adjusted for age and sex, the suburban setting was associated with lower odds of prediabetes or diabetes compared with the urban setting (OR = 0.73, 95% CI 0.57-0.93, p = 0.011), while the rural setting was not significantly associated (OR = 0.81, 95% CI 0.62-1.07, p = 0.139). Older age and male sex were associated with higher odds (age: OR = 1.06, 95% CI 1.06-1.07, p < 0.001; male: OR = 1.27, 95% CI 1.02-1.58, p = 0.031).

After additional adjustment for BMI (Model 2 in Table 5), the suburban association remained present (OR = 0.70, 95% CI 0.54-0.89, p = 0.005), and the rural setting showed lower odds of prediabetes or diabetes that did not reach statistical significance (OR = 0.76, 95% CI 0.57-1.01, p = 0.057). BMI was associated with higher odds (OR = 1.14, 95% CI 1.11-1.18, p < 0.001), while the association with male sex was no longer significant (p = 0.836).

Finally, in the fully adjusted model (Model 3 in Table 6), which additionally included alcohol consumption and regular exercise, the suburban setting remained associated with lower odds (OR = 0.69, 95% CI 0.54-0.89, p = 0.005), while the rural setting was not significant (OR = 0.77, 95% CI 0.58-1.02, p = 0.067). Age and BMI remained associated with higher odds, and lower odds were observed for occasional (OR = 0.66, 95% CI 0.51-0.86, p = 0.002) and daily alcohol consumption (OR = 0.46, 95% CI 0.35-0.64, p < 0.001) compared with rarely consuming alcohol. Regular exercise and sex were not significantly associated. 

A sensitivity analysis additionally adjusting for hypertension did not change the results (suburban OR = 0.69, 95% CI 0.54-0.89; p = 0.005; Appendix 2).

## Discussion

This study investigated the association between geographical living settings and the prevalence of prediabetes or diabetes in participants of northern Ibaraki and examined related health-related lifestyle behaviors and sociodemographic factors such as sex and age. Suburban settings were associated with lower odds of prediabetes or diabetes compared with urban settings, even after adjusting for other factors. Rural areas also showed lower odds, but not statistically significant.

At the descriptive level, the crude prevalence of prediabetes or diabetes was similar across the three geographical living settings. This apparent similarity, however, deserves a closer look, since suburban participants had the highest mean age and the highest prevalence of hypertension among the three settings, characteristics that would usually be expected to increase, rather than reduce, the prevalence of prediabetes or diabetes. Because the suburban association reached significance only after adjustment, it appears that the older age and overall health characteristics of suburban residents had concealed this pattern in the raw data, rather than creating it. In other words, the result seems to reflect something about the suburban setting itself, rather than the individual characteristics of its residents.

These findings are consistent with studies from China and India, where rural areas have been reported to have a lower occurrence of diabetes than more urbanized areas [25,26]. In contrast, studies from the United States and Australia have reported higher rates of diabetes and poorer diabetes-related outcomes in rural areas [27,28], often linked to socioeconomic disadvantage, remoteness, and limited access to health care [29,30]. A possible explanation for these contrasting results is the difference in healthcare access across countries. In Japan, where universal health coverage and shorter distances to services reduce such obstacles even in less urbanized areas, rural residence may not carry the same disadvantage, which may help explain why lower odds were observed outside the urban setting in this study.

Suburban settings may combine relatively good access to healthcare and services typical of more densely populated areas with lower stress and better access to green spaces [31,32]. These characteristics may contribute to the lower odds observed in suburban participants, although their specific roles could not be assessed in this study.

Daily and occasional alcohol consumption were associated with lower odds of prediabetes or diabetes compared with rare consumption. However, this finding should be interpreted with caution. First, the cross-sectional design does not allow causal interpretation. Secondly, the alcohol variable used in all analyses reflected only how often alcohol was consumed, not the amount, the type, or whether it was consumed with or without food. A sensitivity analysis, additionally adjusting for hypertension, did not change the results; however, other unmeasured factors may still have influenced this association.

The association between alcohol consumption and lower odds of prediabetes or diabetes in this study is consistent with a possible protective association between moderate alcohol intake and glycemic status, as reported in other observational and cross-sectional studies [33]. Experimental and observational evidence has linked moderate alcohol consumption to improved insulin sensitivity and anti-inflammatory responses, but the relationship varies across populations and is still not fully understood [34,35]. Some studies have reported lower odds of type 2 diabetes with moderate consumption of alcoholic beverages, particularly when low-grade alcoholic drinks are taken with food [36,37], while high alcohol consumption has been associated with less favorable findings in others [38]. Studies in Japanese male populations have shown inconsistent results. Some suggested a possible protective association with moderate intake, and others did not find such a relation, while indicating that high alcohol consumption may have different implications in Japan than in other countries, possibly due to genetic and lifestyle differences particular to Japanese populations [39,40]. In this study, this association should be interpreted carefully, as reverse causation cannot be ruled out, since healthier participants or those with better health conditions may tend to consume alcohol moderately or daily, in comparison to participants with compromised health, who might be inclined to reduce alcohol intake and have further health consciousness.

Several studies have noted that differences between populations and methods may contribute to contradictory results in the relationship between alcohol consumption and glycemic status [41,42], and substantial evidence supports the negative impact of heavy alcohol consumption on health outcomes, with public health practices and policies stating the benefits of reduced alcohol intake and promotion of healthy behaviors.

Regular exercise was not significantly associated with the lower prevalence of prediabetes or diabetes in this study, which differs from findings in previous studies. Regular exercise has been associated with anti-inflammatory effects and improved blood glucose control through molecular and biochemical mechanisms [43,44], and has been linked to lower odds of type 2 diabetes and cardiovascular disease, as well as to slowing their progression [44,45]. Differences in the type, duration, and intensity of physical activity, as well as the role of dietary habits, may also shape these associations [46,47]. The absence of a significant association observed in this study may partly reflect contextual differences in how physical activity was performed across geographical living settings, for example, higher levels of occupational physical activity in rural and agricultural areas compared with more structured leisure exercise in urban settings [48,49], which may not have been adequately captured by the regular exercise variable used in the present study, as this variable reflected structured exercise rather than activity done through work or daily routines.

Health-related lifestyle behaviors differed significantly between males and females, which is consistent with previous research [50,51]. In this study, males showed higher odds of prediabetes or diabetes compared with females before further adjustments, but this association was no longer significant after adjusting for BMI and the two lifestyle behaviors selected. This suggests that the differences in glycemic status between males and females may be, in some part, explained by differences in BMI and lifestyles rather than by sex alone. Sex-related lifestyle differences may also interact with the living setting, as cultural and occupational roles tend to differ between men and women across rural, suburban, and urban areas, contributing to the varying results observed across settings.

The strengths of this study first lie in the use of data from a community-based cohort in which glycemic status was determined via objective measurements, including HbA1c, obtained during actual health examinations rather than through self-report of prediabetes or diabetes. The large sample size for a regional study (n = 1880) allowed for adjusted analyses accounting for age, sex, BMI, and selected lifestyle behaviors. Secondly, the inclusion of suburban areas as a distinct category alongside rural and urban settings allowed comparisons beyond the simple urban-rural approach widespread in the literature, capturing the characteristics of an intermediate living environment that is often overlooked [52]. Thirdly, this study was conducted in a regional area of Japan, centered on a mid-sized prefectural capital with approximately 270000 residents, rather than one of the country's major metropolitan centers [23], where the influence of living settings on glycemic status has received relatively less attention. Finally, the robustness of the main finding was supported by sensitivity analyses adjusting for hypertension and examining the diabetes and prediabetes categories separately, which left the results essentially unchanged (Appendix 2 to Appendix 4).

Some limitations emerge in this study. Primarily, the cross-sectional design does not allow causal relationships or the directionality of associations to be determined, and exact residential addresses were not available, so geographical areas needed to be assigned from the outreach examination sites as a representative of residence, which may have introduced some misclassification. Additionally, the lifestyle estimations were limited to self-reported questionnaire data, which may be subject to recall and reporting bias, and did not capture occupational physical activity or detailed information on alcohol consumption, such as type. Finally, socioeconomic factors were not available for adjustment, so residual confounding cannot be completely excluded. In addition, as participants attended annual community health examinations, they may represent a relatively health-conscious portion of the population; however, these examinations are conducted as part of a community health program organized in partnership with JA Ibaraki [20,53], not as a clinical service for patients seeking care, which may limit, though not eliminate, their impact.

These findings emphasize the complex relationship between geographical living settings, health-related lifestyle behaviors, and demographic factors in shaping the prevalence of prediabetes or diabetes. Further studies examining the influence of living settings on glycemic status are needed to confirm and extend these findings.

## Conclusions

Suburban living settings were associated with lower odds of prediabetes or diabetes compared with urban settings, even after adjustment for age, sex, BMI, and lifestyle behaviors. Rural areas also showed lower odds, although this association did not reach statistical significance. The crude prevalence of prediabetes or diabetes did not differ significantly across settings, and the lower odds in suburban areas emerged only after adjustment, indicating that this association was initially masked by the demographic characteristics of suburban participants rather than explained by them. These findings highlight the potential role of living environments in influencing the prevalence of prediabetes or diabetes and support the inclusion of suburban settings as a distinct category in future research and public health strategies.

## Appendices

Appendix 1 

**Table 7:** Health-related lifestyle behaviors questionnaire.

Selected questions		Answer
1.	Do you use antihypertensive drugs?	Yes/No
2.	Do you use insulin injections or other antidiabetic drugs?	Yes/No
3.	Have you smoked tobacco in the last month?	Yes/No
4.	Have you gained 10 kg or more since your 20s?	Yes/No
5.	Do you usually exercise for more than 30 minutes, more than two times per week, or for more than one year?	Yes/No
6.	Do you walk daily or do other similar physical activities for more than one hour per day?	Yes/No
7.	Do you walk faster than other people of similar age as yours?	Yes/No
8.	Do you eat faster, normally, or slower than others?	Fast/Normal/Slow
9.	Do you usually have dinner within two hours before going to sleep more than three times a week?	Yes/No
10.	Do you have snacks or sweet drinks in addition to the normal three meals (breakfast, lunch, and dinner)?	Everyday/Sometimes/Rarely
11.	Do you tend to skip breakfast more than three times a week?	Yes/No
12.	How often do you drink alcoholic beverages (such as beer, wine, whisky, sake, etc.)?	Daily/Occasionally/Rarely
13.	Do you sleep enough during the night?	Yes/No

Appendix 2

**Table 8:** Sensitivity analysis of the association between geographical living setting and the prevalence of prediabetes or diabetes (n = 1870), additionally adjusted for hypertension. Model 3 plus hypertension: missing alcohol consumption, n = 9; regular exercise, n = 2; hypertension, n = 1; 10 cases excluded, n analyzed = 1870. Abbreviations: CI, confidence interval; df, degrees of freedom; F, Wald F test; OR, odds ratio; χ², Wald chi-square test. ^a^Wald chi-square was used for categorical variables, whereas Wald F was used for continuous variables. ^b^Hypertension: SBP ≥140 mmHg and/or DBP ≥90 mmHg and/or use of antihypertensive medication. *p < 0.05.

Variables	OR	95% CI	Test statistic^a^	df	p-value
Area					
Urban	Reference				
Suburban	0.692	0.536-0.893	χ² = 8.02	1	0.005*
Rural	0.767	0.577-1.020	χ² = 3.32	1	0.068
Age (continuous)	1.062	1.053-1.071	F = 195.84	1	0.000*
Sex					
Female	Reference				
Male	1.151	0.896-1.479	χ² = 1.21	1	0.271
BMI (continuous)	1.133	1.097-1.171	F = 58.01	1	0.000*
Alcohol consumption					
Rarely	Reference				
Occasionally	0.660	0.508-0.857	χ² = 9.73	1	0.002*
Daily	0.459	0.336-0.627	χ² = 24.03	1	0.000*
Regular exercise					
No	Reference				
Yes	1.201	0.904-1.597	χ² = 1.60	1	0.206
Hypertension^b^					
No	Reference				
Yes	1.191	0.922-1.538	χ² = 1.79	1	0.181

Appendix 3 

**Table 9:** Sensitivity analysis of the association between geographical living setting and the prevalence of diabetes (HbA1c ≥ 6.5% or antidiabetic medication) compared with the normoglycemic group (n = 808), adjusted for age, sex, BMI, alcohol consumption, and regular exercise. Abbreviations: CI, confidence interval; df, degrees of freedom; F, Wald F test; OR, odds ratio; χ², Wald chi-square test. ^a^Missing alcohol consumption, n = 5; regular exercise, n = 1; 5 cases excluded, n analyzed = 803. ^b^Wald chi-square was used for categorical variables, whereas Wald F was used for continuous variables. *p < 0.05.

Variables	OR	95% CI	Test statistics^b^	df	p-value
Area					
Urban	Reference				
Suburban	0.857	0.513-1.433	χ² = 0.34	1	0.557
Rural	0.902	0.493-1.650	χ² = 0.11	1	0.737
Age (continuous)	1.109	1.089-1.130	F = 121.42	1	<0.001*
Sex					
Female	Reference				
Male	2.276	1.333-3.887	χ² = 9.08	1	0.003*
BMI (continuous)	1.361	1.272-1.455	F = 80.34	1	<0.001*
Alcohol consumption^a^					
Rarely	Reference				
Occasionally	0.382	0.207-0.706	χ² = 9.44	1	0.002*
Daily	0.334	0.181-0.615	χ² = 12.4	1	<0.001*
Regular exercise^a^					
No	Reference				
Yes	1.36	0.799-2.313	χ² = 1.28	1	0.257

Appendix 4 

**Table 10:** Sensitivity analysis of the association between geographical living setting and the prevalence of prediabetes (HbA1c 5.6-6.4% without antidiabetic medication) compared with the normoglycemic group (n = 1697), adjusted for age, sex, BMI, alcohol consumption, and regular exercise. Abbreviations: CI, confidence interval; df, degrees of freedom; F, Wald F test; OR, odds ratio; χ², Wald chi-square test. ^a^Missing alcohol consumption, n = 7; regular exercise, n = 2; eight cases excluded, n analyzed = 1689. ^b^Wald chi-square was used for categorical variables, whereas Wald F was used for continuous variables. *p < 0.05.

Variables	OR	95% CI	Test statistics^b^	df	p-value
Area					
Urban	Reference				
Suburban	0.678	0.524-0.878	χ² = 8.70	1	0.003*
Rural	0.765	0.574-1.020	χ² = 3.33	1	0.068
Age (continuous)	1.06	1.051-1.068	F = 121.42	1	<0.001*
Sex					
Female	Reference				
Male	1.118	0.868-1.439	χ² = 0.75	1	0.387
BMI (continuous)	1.119	1.084-1.155	F = 48.26	1	<0.001*
Alcohol consumption^a^					
Rarely	Reference				
Occasionally	0.700	0.539-0.910	χ² = 7.11	1	0.008*
Daily	0.482	0.352-0.659	χ² = 20.83	1	<0.001*
Regular exercise^a^					
No	Reference				
Yes	1.175	0.881-1.566	χ² = 1.21	1	0.272
